# MiR-let-7a inhibits cell proliferation, migration, and invasion by down-regulating PKM2 in gastric cancer

**DOI:** 10.18632/oncotarget.6821

**Published:** 2016-01-05

**Authors:** Ran Tang, Chao Yang, Xiang Ma, Younan Wang, Dakui Luo, Chi Huang, Zekuan Xu, Ping Liu, Li Yang

**Affiliations:** ^1^ Department of General Surgery, the First Affiliated Hospital of Nanjing Medical University, Nanjing, China; ^2^ Liver Transplantation Center of the First Affiliated Hospital and Key Laboratory on Living Donor Liver Transplantation, Ministry of Health, Nanjing Medical University, Nanjing, China; ^3^ Department of Oncology, the First Affiliated Hospital of Nanjing Medical University, Nanjing, China

**Keywords:** gastric cancer, microRNA-let-7a, PKM2

## Abstract

In contrast to normal differentiated cells that depend on aerobicoxidation for energy production, cancer cells use aerobic glycolysis as the main source (Warburg's effect). The M2 splice isoform of pyruvate kinase (PKM2) is the key regulator for the aerobic glycolysis, high expression of PKM2 contributes to the aerobic glycolysis, promotes the growth of tumors. In the present study, we found that PKM2 was highly expressed in gastric cancer (GC) tissues and had a strongly inverse correlation with the expression of microRNA-let-7a (miR-let-7a). Furthermore, we found that the overexpression of miR-let-7a markedly suppressed the proliferation, migration, and invasion of GC cells by down-regulating the expression of PKM2. MicroRNAs (miRNAs) are important regulators play key roles in tumorigenesis and tumor progression. Although previous reports showed that let-7 family members act as tumor suppressors in many cancers. The specific regulatory mechanism of miR-let-7a to PKM2 in gastric cancer is still unclear. In this study, we revealed that miR-let-7a function as the antitumor and gene regulatory effects of PKM2 in GC cells.

## INTRODUCTION

Gastric cancer (GC) is the fourth most common lethal neoplasm in the world, especially in Eastern Asia, Eastern Europe and South America. GC is also the second most common cause of death from cancer [1]. In the past few years, with advances in surgical techniques, chemical therapy, radiotherapy and molecular targeted therapy to cancers, the prognosis of GC was improved [2], but the long-term outcomes of GC patient remained dismal, especially for advanced GC with a 5-year overall survival rate of 25% or less [3]. Recently, more and more miRNAs, oncogenes and tumor suppressor genes have been confirmed to be closely associated with GC, but the specific molecular mechanisms on the proliferation, migration and invasion of the cancer cells have been still under investigation.

Aerobic glycolysis is the common feature of cancer cells, tumor cells showed high glycolytic rate with production of lactate even in an oxygen-rich condition - a phenomenon firstly reported by Otto Warburg [4]. The M2 splice isoform of pyruvate kinase (PKM2) is a key regulator of aerobic glycolysis as an enzyme that catalyzes the later step of glycolysis. As the four known isoforms of pyruvate kinase, PKM1 and PKM2 derive from an alternative splicing of the same PKM2 gene [5]. PKM1 is expressed in the skeletalmuscle and brain; In addition to embryonic cells and adults stem cells, PKM2 is also a major isoform expressed in cancer cells [6, 7]. The switch of PKM1 to the PKM2 isoform, which observed in different tumors [8, 9], has been shown to be essential for the “Warburg effect” and the growth of the tumor [10].

Heterogeneous nuclear ribonucleoproteins (hnRNP) I, A1, and A2 are bound to RNA sequences encoded by exon 9 and inhibit PKM1 mRNA splicing. The oncoprotein c-Myc activates transcription of hnRNPI (PTB), hnRNPA1, and hnRNPA2, resulting in preferential PKM2 isoform expression [11]. C-Myc/hnRNPA1/PKM2 may play an important role in the growth of GC, but the molecular mechanisms that regulate this signal pathway need further exploration.

Increasing evidence has indicated that miRNAs are involved in the development and progression of cancers, acting as tumor suppressors or oncogenes [12]. MiR-let-7a which was first identified in C.elegans as a heterochronic gene, promotes the transition of larval stage 4-to-adult [13]. Further research on miR-let-7a revealed its function in cell proliferation, differentiation, apoptosis, and metabolism [14, 15]. The miR-let-7a level were found to be low in different human cancers, and its loss or down-regulation was associated with increased cancer aggressiveness and poor clinical outcome [16, 17]. Ectopic expression of let-7a reduced chemoresistance and invasiveness of cancer cells, suppressing tumor growth of human lung cancer *in vivo* [18]. Previous study found that let-7a functioned as a tumor suppressor by targeting the oncogene c-Myc [19, 20].

Our work revealed that miR-let-7a was significantly down-regulated in human gastric cancer specimens and inhibited the growth, migration, invasion and tumorigenicity of GC cell *in vitro* and vivo. Further data showed the expression of miR-let-7a was negatively correlated with the expression of c-Myc, hnRNPA1 and PKM2. To our knowledge, our data is the first report showing that miR-let-7a regulates the expression of PKM2 through c-Myc and hnRNPA1 in GC cells. The results identifying a new signal pathway miR-let-7a/c-Myc/hnRNPA1/PKM2 suppresses the growth and proliferation of gastric cancer, providing new insights into the pathogenesis of gastric cancer and evolvable the therapeutic strategies.

## RESULTS

### miR-let-7a expression is negatively correlated with the PKM2 levels in both gastric cancer tissues and cell lines

**Figure 1:**
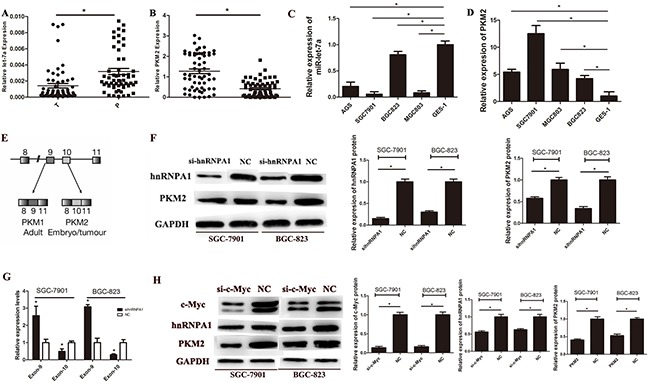
The expression of miR-let-7a and PKM2 in gastric cancer tissues and cell lines **A.** The expression of miR-let-7a in GC tissues and paired adjacent non-tumor tissues were detected by RT-PCR assay, U6 was used as an internal control. **B.** The expression levels of PKM2 in GC tissues and non-GCs were measured by RT-PCR. PKM2 expression was normalized by β-actin. **C.** The expression levels of miR-let-7a were measured in gastric cancer cell lines and normal gastric mucosa epithelial cell line (GES-1) by RT-PCR. **D.** The expression levels of PKM2 were measured in gastric cancer cell lines and normal gastric mucosa epithelial cell line (GES-1) by RT-PCR. **E.** Schematic diagram of PKM splicing. **F.** Westernblotting identified hnRNPA1 and PKM2 expression changed following transfection of SGC-7901 and BGC-823 with si-hnRNPA1.GAPDHused as a normal control. **G.** The exon 9 up-regulated while exon 10 was down-regulated in si-hnRNPA1-transfected gastric cancer cells. **H.** Westernblotting identified hnRNPA1 and PKM2 expression changed following transfection of SGC-7901 and BGC-823 with si-c-Myc. GAPDH was used as a normal control. Asterisks means a significant difference compared with the controls (*P*<0.05).

To determine whether miR-let-7a expression correlates with the levels of PKM2 in GC tissues and cell lines. Sixty pairs of gastric cancer (GC) tissues and their adjacent normal gastric tissues (NG) were used to determine the expression levels of miR-let-7a and PKM2 by real-time polymerase chain reaction (RT-PCR). As shown in Figure 1A and 1B, the expression level of miR-let-7a was significant down-regulated in GC tissues comparedwith the adjacent normal tissues (*p* = 0.0002), while the expression levels of PKM2 was dramatically higher in GC tissues than that in the adjacent normal tissues (p<0.0001). Among the 60 pairs of tissues, 47/60 (78.3%) showed miR-let-7a down-regulated (T/N<1.0), and 13/60 (21.6%) were upregulated (T/N>1.0). For PKM2, 52/60(86.6%) were elevated in GCs compared with the adjacent normal tissues. The same results were showed in the GC cells (Figure 1C and 1D). These results indicated that miR-let-7a expression was down-regulated in both GC tissues and GC cell lines and negatively correlated with the levels of PKM2. Furthermore, we assessed the correlation between miR-let-7a or PKM2 expression levels and clinicopathological features. As shown in Table 1, miR-let-7a was lower in tissues with poorly and moderately differentiated type, lymph node metastasis N1-N3 and stage III-IV. The level of PKM2 was associated with histological type and lymphatic invasion.

**Table 1:** Expression of miR-let-7a and PKM2 in human gastric cancer according to clinicopathological features of patients

Clinicopathological variables	(miR-let-7a T>N (*n* = 13)	Expression) T<N (*n* = 47)	*P* value	(PKM2 T>N (*n* = 52)	Expression) T<N (*n* = 8)	*P* value
Age(year)						
<50	5	22		28	5	
>50	8	25	0.755	24	3	0.719
Gender						
Male	7	27		30	3	
Female	6	20		22	5	0.448
Tumor size(cm)						
<3	3	23		25	2	
≥3	10	24	0.122	27	6	0.276
Histological type						
Well	6	7		12	6	
Moderately and poorly	7	40	0.025*	40	2	0.007*
Depth						
Localized in subserosa	8	20		32	4	
Beyond subserosa	5	27	0.34	20	4	0.702
Lymph node metastasis						
N0	9	16		19	5	
N1-N3	4	31	0.03*	33	3	0.456
Lymphatic invasion						
Absent	6	19		30	1	
Present	7	28	0.758	22	7	0.024*
Stage						
I, II	8	12		10	3	
III, IV	5	35	0.022*	42	5	0.353

* *P*<0.05 statistically significant difference

Taken together, these data provided evidence that miR-let-7a plays an important role in the pathogenesis of GC by regulating the expression of PKM2.

### HnRNPA1 direct regulates the expression of PKM2 in gastric cancer cells

HnRNPA1 promotes the generation of PKM2 by bindings repressively to exon 9 (Figure 1E) [11]. To confirm the function of hnRNPA1 on the expression of PKM2, we used small interfering RNA (siRNA) to knockdown the expression of hnRNPA1 in SGC-7901 and BGC-823. Unsurprisingly, down-regulated hnRNPA1 led to a decreased PKM2 expression (Figure 1F). Then we designed specific primers for exon 9 and exon 10. Results of RT-PCR showed that the exon 9 was up-regulated while exon 10 was down-regulated in si-hnRNPA1-transfected gastric cancer cells (Figure 1G). Our data indicates hnRNPA1 directly regulates the expression of PKM2 in gastric cancer cells.

### C-Myc regulates PKM2 by enhancing the transcription of hnRNPA1

The putative c-Myc binding sites located at E boxes(CACGTG) within a ∼700nt hnRNPA1 promoter region, and c-Myc activates transcription of hnRNPI (PTB), hnRNPA1, and hnRNPA2, resulting in preferential PKM2 isoform expression [11]. To further explore the direct relationship between c-Myc and hnRNPA1 in gastric cancer cells, siRNA was used to down-regulate the expression of c-Myc in SGC-7901 and BGC-823, the results showed that the down-regulation of c-Myc led to the inhibition of hnRNPA1, furthermore, the expression of PKM2 also significantly decreased (Figure 1H). These data confirmed that c-Myc indirectly regulates PKM2 by enhancing the transcription of hnRNPA1 in gastric cancer cells.

### miR-let-7a interfere the expression of c-Myc/hnRNPA1/PKM2 in gastric cancer cells

**Figure 2:**
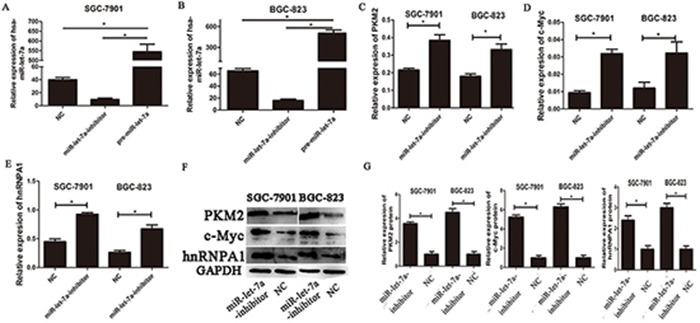
The expressions of c-myc/hnRNPA1/PKM2 were up-regulated in gastric cancer cells transfected with the miR-let-7a-inhibitor **A–B.** SGC-7901 and BGC-823 were transfected with specific miR-let-7a inhibitor, pre-miR-let-7a respectively. The cells transfected with empty lentiviral construct vectors as negative control (NC). The expression of miR-let-7a was analyzed by miRNA RT-PCR after the transfection, the cells transfected with the empty lentiviral vectors were used as negative control. **C–E.** The expression levels of PKM2, c-Myc and hnRNPA1 in the cells after the transfection of miR-let-7a-inhibitor were detected by qRT-PCR, the cells transfected with the empty lentivival vectors were used as negative control. **F–G.** The expression levels of PKM2, c-Myc and hnRNPA1 in the miR-let-7a-inhibitor group and negative control group were analyzed by western blotting. Asterisks means the significant difference compared with the controls (*P*<0.05).

**Figure 3:**
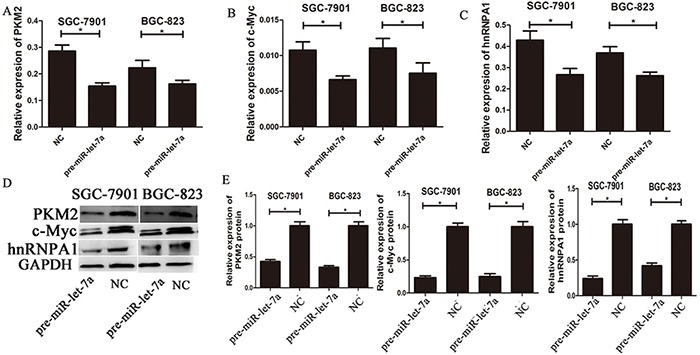
The expressions of c-myc/hnRNPA1/PKM2 were down-regulated by overexpression of miR-let-7a **A–C.** The expression levels of PKM2, c-Myc and hnRNPA1 in the GC cells transfected with the miR-let-7a mimics (pre-miR-let-7a) were detected by qRT-PCR, the cells transfected with the empty lentiviral vectors were used as negative control. **D–E.** The expression levels of PKM2, c-Myc and hnRNPA1 in the pre-miR-let-7a group and negative control group were analyzed by western blotting. Asterisks means the significant difference between miR-let-7a-inhibitor or pre-miR-let-7a group compared with negative control, respectively; (*P*<0.05).

To investigate the impact of miR-let-7a on PKM2 levels in GC cell lines, we used lentiviral transfection to construct miR-let-7a overexpression (pre-miR-let-7a) and knockdown (miR-let-7a-inhibitor) GC cell lines including SGC-7901 and BGC-823. Figure 2A and 2B, which showed the expression of miR-let-7a in GC cell SGC-7901 and BGC-823 after lentiviral transfection. Then the expression of PKM2 and c-Myc, hnRNPA1 were detected by RT-PCR and western blotting. Notably, compared to the negative control group (NC), the inhibitor of miR-let-7a resulted in the significantly upregulation of PKM2 (*p* = 0.021 and *p* = 0.011 for SGC-7901 and BGC-823 separately) and c-Myc (*p* = 0.001 and *p* = 0.049 separately), hnRNPA1 (p<0.001 and *p* = 0.06 separately), (Figure 2C–2G). Conversely, they were all significantly reduced in GC cell lines with the overexpression of miR-let-7a (Figure 3), *p* = 0.07 and 0.03 separately for PKM2; *p* = 0.032 and 0.041 separately for c-Myc; *p* = 0.036 and 0.034 separately for hnRNPA1. These results indicated that miR-let-7a interfered the pathway of c-Myc/hnRNPA1/PKM2 in gastric cancer cells, and miR-let-7a inhibited the expression of PKM2 by regulating the expression of c-Myc and hnRNPA1.

### miR-let-7a inhibit cellular proliferation and colony formation

**Figure 4:**
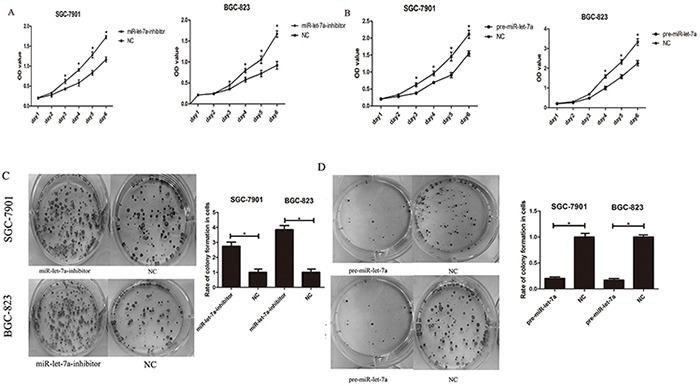
miR-let-7a inhibited the proliferation of the GC cells SGC-7901 and BGC-823 *in vitro* **A–B.** The effects of miR-let-7a on SGC-7901 and BGC-823 cells viability were analyzed by CCK-8 assay at the indicated time point of everyday after transfection with pre-miR-let-7a or miR-let-7a inhibitor, with empty vector as negative control respectively. **C–D.** Effects of miR-let-7a on the colony formation of SGC-7901 and BGC-823 were shown. The crystal violet-stained cell colonies were calculated at day 14 after transfection. Asterisks means the significant difference between miR-let-7a-inhibitor or pre-miR-let-7a group compared with negative control respectively. The data was displayed as mean ±SD, *P*<0.05.

To confirm the hypothesis that miR-let-7a functions as a potential suppressor in GC cells, we also investigated the influence of miR-let-7a expression on cellular growth and colony formation. Here CCK-8 assay were performed to observe the proliferation of SGC-7901 and BGC-823 in different groups (miR-let-7a inhibitor, pre-miR-let-7a and negative control). As shown in Figure 4A, the proliferation of GC cells in miR-let-7a-inhibitor group was significantly increased compared with the control group (*p* = 0.021). By contrast, the GC cells which were transfected with pre-miR-let-7a caused a significant decrease in proliferation compared with the control group (Figure 4B), *p* = 0.038.

Furthermore, to evaluate the long-term effects of miR-let-7a on cell proliferation, the colony formation assay was employed. Our results showed that the down-regulation of miR-let-7a significantly enhanced the colony formation ability of SGC-7901 and BGC-823 compared with the negative control (Figure 4C), *p* = 0.025 and 0.034 separately. Conversely, fewer colonies were formed in GC cells transfected with pre-miR-let-7a compared with the negative control (Figure 4D), *p* = 0.037 and 0.036 separately.

Our results suggested that miR-let-7a negatively regulates the proliferation and colony formation of GC cells *in vitro*.

### miR-let-7a is involved in the negative regulation of GC cell migration and invasion

**Figure 5:**
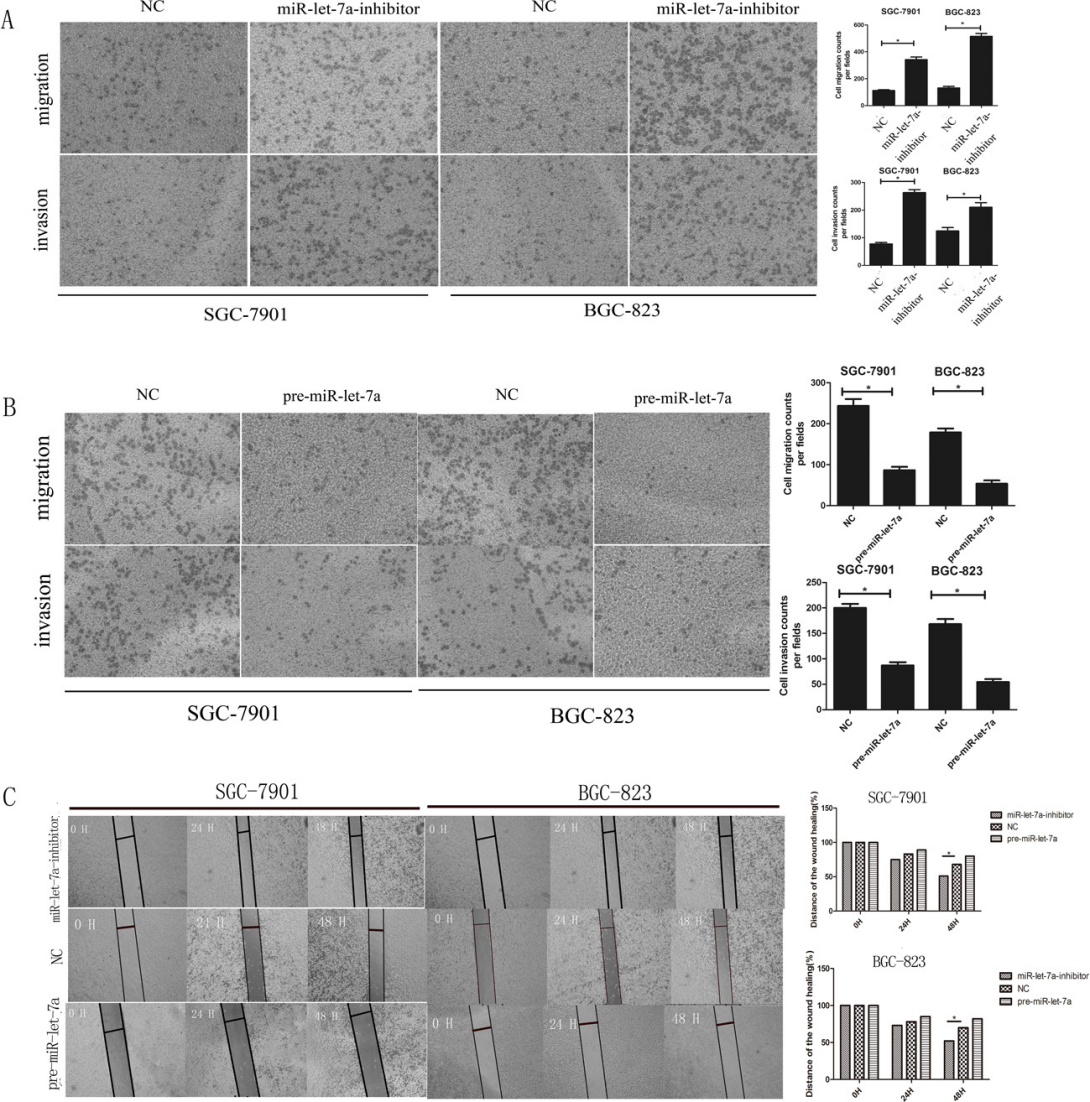
miR-let-7a negatively regulated the migration and invasion ability of SGC-7901 and BGC-823 *in vitro* **A–B.** Transwell migration and Matrigel invasion assays were performed in the SGC-7901 and BGC-823 cells after being transfected with pre-miR-let-7a, miR-let-7a inhibitor and empty vector. Compared with the negative control, down-regulation of miR-let-7a increased the ability of migration and invasion of SGC-7901 and BGC-823. Overexpression of miR-let-7a significantly reduced the number of GC cells that migrated or invaded to the lower surface of membrane. **C.** Wound healing assay was used to SGC-7901 and BGC-823 after being transfected with pre-miR-let-7a, miR-let-7a-inhibitor and empty vectors, relative ratio of wound closure per field was shown. Image J Plus was used to analyze the results. All data used t test and was shown as mean±SD, *P<0.05.

To confirm that miR-let-7a function as a tumor suppressor in GC, we investigated the influence of miR-let-7a on the migration and invasion of GC cells *in vitro*. We used cell migration and Matrigel invasion assays to investigate the effects of miR-let-7a on GC cells’ migration and invasion. Our data indicated that the ability of migration and invasion of SGC-7901 and BGC-823 were enhanced obviously when miR-let-7a down-regulated in SGC-7901 and BGC-823 (Figure 5A), p<0.001 for migration of SGC-7901 and BGC-823. On the contrary, migration and invasion of GC cells were significant suppressed by the overexpression miR-let-7a (Figure 5B), both p<0.001.

Wound healing assays also supported the conclusion that miR-let-7a suppressed the migration ability of GC cells. Results confirmed that the miR-let-7a-inhibitor significantly accelerated the cell migration of the GC cells. Meanwhile, the pre-miR-let-7a was distinctively less migratory (Figure 5C).

### miR-let-7a suppresses the tumorigenicity *in vivo*

**Figure 6:**
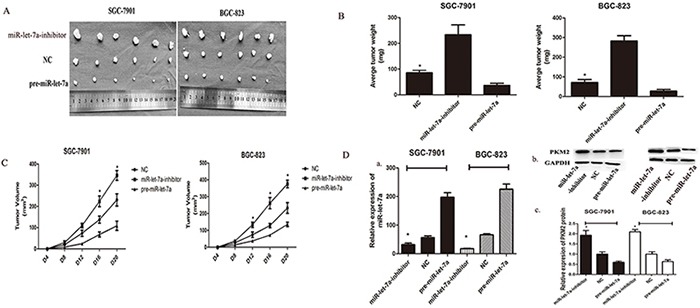
miR-let-7a inhibited xenograft tumor growth of GC cells SGC-7901 and BGC-823 **A.** GC cells SGC-7901 and BGC-823 were transfected with pre-miR-let-7a showed the significant inhibition to the tumor formation in the flank of nude mice. Cells transfected with empty vectors were used as negative control. The promotion effects were observed in the GC cells transfected with miR-let-7a-inhibitor. **B–C.** The graphs represented the growth of tumors 21 days after inoculation. The weight and volume of tumors were calculated. **D.** The expression of PKM2 and miR-let-7a in the implanted tumors were evaluated by western blotting and miRNA RT-PCR, and all data are shown as mean±SD. *P<0.05

To assess the effects of miR-let-7a on tumorigenicity *in vivo*, transfected GC cells were injected into the flanks of nude mice. The cells transfected with negative miR-let-7a lentiviral vector which was used as negative control were injected into the opposite flank of the same mice. After 28 days, we observed the tumor size, notably, miR-let-7a silencing significantly promoted tumorigenicity *in vivo* as the tumor whose size was significantly increased. Meanwhile, the tumors of pre-miR-let-7a group were significantly smaller than that of miR-let-7a-NC group (Figure 6A). The expression of miR-let-7a and PKM2 in the implanted tumors were evaluated by miRNA RT-PCR (*p<0.05) and western blotting (*p<0.05) in Figure 6B and 6D.

Growth curves of the implanted tumors supported the conclusion that miR-let-7a inhibits tumor growth *in vivo* (Figure 6C) *p* = 0.03 for SGC-7901, *p* = 0.013 for BGC-823. We also found that the average weight of the implanted tumors from the miR-let-7a-inhibitor group was significantly heavier than that in the control group. Conversely, tumors from the pre-miR-let-7a group were lighter than that in the control group (Figure 6D), *p* = 0.02 and 0.017 separately for miR-let-7a-inhibitor and pre-miR-let-7a of SGC-7901; P=0.002 and 0.36 separately for miR-let-7a-inhibitor and pre-miR-let-7a of BGC-823.

Taken together, miR-let-7a could inhibit the tumor growth *in vivo*.

### PKM2 is involved in the regulation of cell proliferation, migration and invasion by miR-let-7a

**Figure 7:**
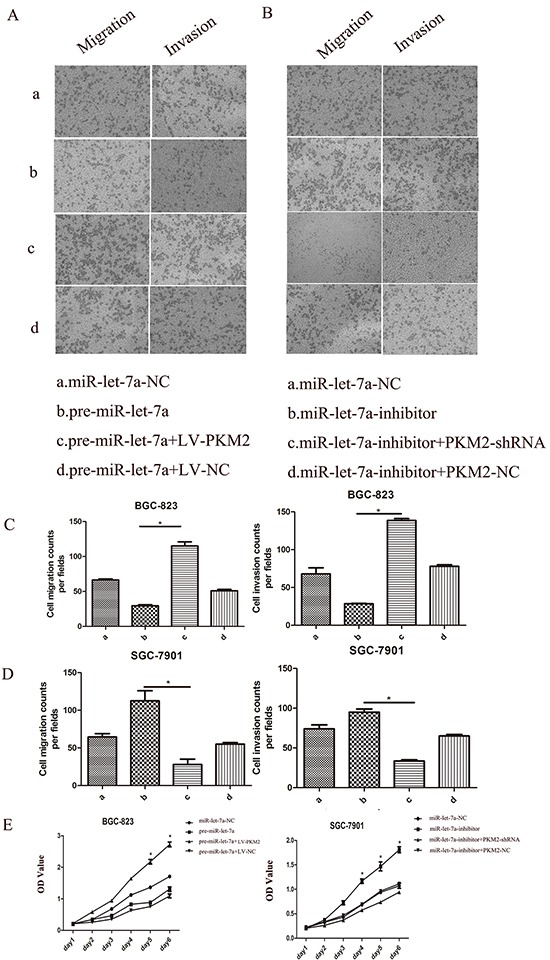
The role of miR-let-7a and PKM2 in the regulation of gastric cancer cell proliferation, migration and invasion BGC-823 (a: miR-let-7a-NC; b: pre-miR-let-7a; c. pre-miR-let-7a+LV-PKM2; d. pre-miR-let-7a+LV-NC). SGC-7901(a: miR-let-7a-NC; b:miR-let-7a-inhibitor; c:miR-let-7a-inhibitor+PKM2-shRNA; d:miR-let-7a-inhibitor+PKM2-NC). **A–E.** Upreguations of let-7a suppressed BGC-823 cell proliferation, migration, invasion. The rescue experiments for miR-let-7a overexpression were performed by ectopic expression of PKM2 (LV-NC). MiR-let-7a silencing was counteracted by downregulation of PKM2 also observed in the SGC-7901 cells. Representative data are displayed as mean±SD values. Asterisks indicate significant differences compared with controls at *P*<0.05.

We have demonstrated that the knockdown of the miR-let-7a promoted the proliferation, migration and invasion of the GC cells and enhanced the expression of the PKM2. By contrast, ectopic miR-let-7a suppressed these phenotypes and PKM2 expression (Figure 2 and 3). To further illustrate that miR-let-7a affects the growth, migration and invasion of GC cells by regulating PKM2, we up-regulated and down-regulated PKM2 expression in GC cells. Subsequently, we investigated whether PKM2 could counteract the inhibition effect induced by miR-let-7a overexpression in GC cells. The vector LV-PKM2, which contained the PKM2 coding sequence, was co-transfected with miR-let-7a precursor and either LV-PKM2 or LV-NC. The results confirmed that ectopic expression of PKM2 reversed the suppression of migration and invasion caused by the overexpression of miR-let-7a (Figure 7A and 7C). In the SGC-7901, the similar rescue effect was also observed in which promotion of cell phenotypes by miR-let-7a silencing was counteracted by downregulation of PKM2 (Figure 7B and 7D). The proliferation of GC cells showed the same effects (Figure 7E).

Taken together, these results are consistent with our hypothesis that miR-let-7a inhibits GC cell proliferation, migration and invasion by regulating PKM2.

## DISCUSSION

Recent studies showed that miRNAs may act as activators or inhibitors of tumor proliferation and metastasis [21]. To date, over 1000 miRNAs have been identified, they had been shown involved in nearly all biological processes. For example, miR-155 can promotes tumor invasion and metastasis in breast cancer [22]. The miR-200 family can inhibit tumor invasion and metastasis through the regulation of EMT [23]. Aberrant expression of miRNAs, abnormal activation of oncogenes or the inactivation of tumor suppressor genes play an important role in the growth of cancer [24]. Recently, aberrant expression, biological functions and related carcinogenesis pathways of miRNAs become a research hotspot.

MiR-let-7a has been identified as tumor suppressor and reported down-regulated in some tumors include GC [25–27]. However, no information is available on the mechanism that altered miR-let-7a affects the growth of gastric cancer cell. In our study, we revealed that miR-let-7a was highly down-regulated in GC tissues and cell lines compared with their nontumorous counterparts or GES-1 cells. We showed that the overexpression of miR-let-7a in GC cell lines resulted in the significantly decreasing in cell proliferation rate, colony formation, migration, invasion *in vitro* and tumorigenicity *in vivo*. On the contrary, miR-let-7a depletion promoted proliferation, colony formation and tumor formation of GC cells. More importantly, we demonstrated that miR-let-7a suppressed the GC cell growth, migration and invasion by down-regulating the expression of PKM2.

PKM2, is the key enzyme of the aerobic glycolysis of the tumor cells [5]. We focused on PKM2 because of its known role as a regulator of glycolysis in malignant tissues. PKM2 expression was shown to be involved in early tumorigenesis [28] and the increasing of PKM2 level correlates with tumor size and stage [7]. Other studies indicated that phosphorylation [29], acetylation [30], and oxidation [31] at the post-transcriptional level affect the expression or function of PKM2 by modulating its enzyme activity or promoting protein degradation. Nevertheless, it is not clear to us, which one of these regulatory mechanisms plays a central role in controlling PKM2 contextually and temporally. In this paper, we reported that miR-let-7a suppressed the cell proliferation, migration and invasion in gastric cancer, moreover, co-expression of miR-let-7a and PKM2 could rescue tumor inhibited by miR-let-7a, indicating that PKM2 is the functional target of miR-let-7a in gastric cancer. However, the specific mechanism underlying how miR-let-7a affects the expression of PKM2 was not clear.

The oncogene c-Myc and hnRNPA1 had been showed have a strong impact on the expression of PKM2 in tumor cells [11]. HnRNPA1 binds to sequences flanking exon 9 repressively, resulting in exon 10 inclusion. The oncogenic transcription factor c-Myc upregulates the transcription of hnRNPA1, ensuring a high ratio of PKM2/PKM1. We found that the overexpression of miR-let-7a resulted in the decrease of c-Myc, hnRNPA1 and PKM2, and inhibited the proliferation, migration, invasion of GC cells both *in vitro* and *in vivo*. Furthermore,our results shows that PKM2 can counteract the inhibition effect induced by miR-let-7a in gastric cancer cells. These results verified the inhibitory effects of let-7a down-regulating PKM2 in the GC cell.

We do know that our study has certain limitations. Our result showed that miR-let-7a inhibited the proliferation, migration, invasion of GC cells by down-regualting the expression of PKM2, but we do not rule out the mechanism of PKM2 affect the growth of GC cells. We will go further in this study.

In conclusion, our study demonstrated that miR-let-7a has a suppressive role in cell growth, migration and invasion of GC cells. In addition, the mechanism of miR-let-7a inhibited the growth of GC cells is related to the regulation of PKM2. We also demonstrated that the expression level of miR-let-7a was significantly lower in gastric cancer tissues than in adjacent normal tissues and inversely correlated with PKM2 levels. It will be valuable to explore whether miR-let-7a could act as a tumor suppressor to be a novel therapeutic target for gastric cancer therapy.

## MATERIALS AND METHODS

### Human tissue samples

Twenty pairs of gastric cancer specimens. Including primary gastric cancer tissues and paired adjacent non-tumor tissues were acquired from the patients with GC who had undergone surgical at The First Affiliated Hospital of Nanjing Medical University, China. The clinicopathologic characteristics of the patients were diagnosed by two professional pathologists independently. All tissues were frozen in liquid nitrogen and stored at −80°C after the surgical removal immediately. The study was approved by the ethic committee of the Nanjing Medical University and received the informed consent of all the patients.

### Cell lines and cell culture

All cell lines include gastric cancer cell lines AGS, SGC7901, BGC823, MGC803 and human normal gastric mucous epithelium cell GES-1 (ATCC, Manassas, VA, USA) were cultured in RMPI 1640 (Hyclone, Logan, UT, USA) medium contained 10% fetal bovine serum (Gbico, Detroit, MI, USA) and antibiotics (100units/ml penicillin G and 100mg/ml streptomycin). All cell lines were maintained in a humidified incubator containing 5% CO_2_ at 37°C.

### RNA extraction and quantitative polymerase chain reaction (qRT-PCR)

Total RNA from cultured cells and frozen tissues were extracted with Trizol reagent (Invitrogen, Carlsbad, CA, USA) according to manufacturer's instruction. To detect the levels of miR-let-7a, we used Taqman miRNA reverse transcription Kit (Invitrogen, Carlsbad, CA, USA) to get cDNA. U6 was used for normalization. The specific primers were as follows: miR-let-7a, forward: 5′-GGTGAGGTAGTAGGTTGTATAGTT-3′, reverse: 5′-CTCGCTTCGGCAGCACATATA-3′; U6, forward: 5′-CTCGCTTCGGCAGCACA-3′, reverse: 5′-AACGCTTCACGAATTTGCGT-3′. The ABI StepOne Plus (Applied Biosystems, Foster City, CA, USA) was used to perform the real-time PCR. All quantification data were normalized by U6 and each RT reaction was run in triplicate. The relative quantification of let-7a was calculated using the 2^−ΔΔCT^ method normalized to the U6 level.

To detect the levels of PKM2 and relative genes, we used SYBR Green Master Mix Kit (Roche, USA) followed the manufacturer's instruction. β-actin was used for normalization. The specific primers were as follows: PKM2, forward: 5′-CTGTGGACTTGCCTGCTGTG-3′, reverse: 5′-TGCCTTGCGGATGAATGACG-3′; c-Myc, forward: 5′-ACCACCAGCAGCGACTCTGA-3′, reverse: 5′-TCCAGCAGAAGGTGATCCAGACT-3′; hnRNPA1, forward: 5′-TCAGAGTCTCCTAAAGAGCCC-3′, reverse: 5′-ACCTTGTGTGGCCTTGCAT-3′; β-actin, forward: 5′-AGAAAATCTGGCACCACACC-3′, reverse: 5′-TAGCACAGCCTGGATAGCAA-3′. All procedures were performed in triplicate.

### Cell transfection

We modified the commercial LV3-hsa-miR-let-7a-pre-microRNA vector, LV3-hsa-miR-PKM2 vector (pre-miR-let-7a, pre-PKM2) and LV3-hsa-let-7a inhibitor vector, LV3-PKM2 inhibitor vector (miR-let-7a inhibitor, PKM2 inhibitor) lentiviral constructs (Genepharma, Shanghai, China) to overexpressed or knockdown miR-let-7a and PKM2 in GC cells. The LV3 empty construct (miR-NC) was served as a negative control. siRNA for hnRNPA1 and c-Myc were constructed by Genepharma, Shanghai, China. All constructed vectors were verified byDNA sequencing. When SGC7901 and BGC823 cells grew to 40-50% confluence, cells were infected with lentiviral vectors miR-NC, pre-miR-let-7a and miR-let-7a inhibitor respectively. The lentiviral vectors were transfected into GC cells with the multiplicity of infection (MOI) of 10 to the SGC7901 and 50 to the BGC823. Next we added polybrene (Genepharma, Shanghai, China) into the cells with 5ug/ml to increase the transfection efficiency. Stable cell lines were selected by using 3ug/ml puromycin (Sigma, USA) for 1 week. After that, cells were analyzed for miR-let-7a expression.

### Western blotting analysis

Total protein was isolated from cells after transfection. The concentration of the protein was measured by BCA protein assay kit (Beyotime, Shanghai, China) followed by the manufacturer's instruction. Samples were electrophoresed by using 12% SDS-PAGE. Then the protein was transferred to the PVDF membranes (Bio-Rad, California, USA). The membranes were incubated with specific primary antibodies (1:1000) in 4°C overnight after blocking in skim milk. Next the blotted membranes were incubated with HRP-conjugated anti-mouse IgG (1:2000) antibodies at room temperature for 2 hours. Proteins were visualized using a detection system of enhanced chemiluminescence (ECL). GAPDH was used as internal reference. Rabbit antibodies to PKM2, c-myc and hnRNPA1 (Cell Signaling Technology, USA), mouse antibodies to GAPDH (Beyotime, Shanghai, China) were used.

### Cell proliferation and plate colony formation assay

Cell proliferation assays were conducted by using Cell Counting Kit-8 (CCK-8) (Beyotime, Shanghai, China) according to the manufacturer's instructions. 2000 cells were seeded into each well of 96-well platewith 100ul RPMI-1640 supplemented with 10% FBS. At the indicated time point of everyday, the medium was exchanged by 110ul RPMI-1640 with CCK-8 (100ul RPMI-1640 and 10ul CCK-8) and the cells were incubated for 2 hours. Then we measured the absorbance for each well at a wavelength of 450nm (OD value) using an auto-microplate reader. Average OD values were used to estimate the number of cells of each group.

Cell colony formation rate was measured by plate colony formation assay. 400 cells were added to each well of a 6-well plate and incubated for about 2 weeks until colony was obviously formed. Then the plate was gently washed and stained with crystal violet. The amount of the colonies was counted by observing the proliferation of single cell.

### Cell migration and wound healing assay

The ability of cell migration was performed by transwells with 8μm pore size (Corning Costar Corp, USA).5×10^4^ cells suspended by RPMI1640 without fetal bovine serum were placed in the top chamber of each insert. 500μl RPMI1640 with 10% fetal bovine serum were added into the lower chamber. After incubating for 24h-48h at 37°C in a 5% CO^2^ humidified incubator,. The cells on the upper surface were removed with a cotton swab and the cells migrated to the bottom side of the membrane cells were fixed with 95% absolute alcohol and stained with crystal violet for 20 min at room temperature. Four random fields of each membrane were imaged under an inverted microscope (Olympus Corp. Tokyo, Japan) and counted for statistical analysis. Each experiment was performed in triplicate.

To further investigate the cell migration, we used wound healing assay. 8×10^5^ cells were seeded in each well of a 6-well plate. After 24h, when the well was almost full of cells, and linear scratch wounds (in triplicate) were created by 200μl pipette tip. Then the plate was washed by PBS for 5-6 times to remove the suspension cell and the cells were maintained in serum-free media. Images were taken at 0h, 24h, 48h at same areas to observe the migrated cells and wound healing and the cells in three wells from each group were quantified. Image J Plus was used to quantify the wound healing assays.

### Cell invasion assay

The invasive ability of the cells was assayed by using Transwells with 8mm pore size (Corning Costar Corp, USA). The Transwells were put into the 24-well plates. First we added 0.1ml Matrigel (50μg/ml, BD Biosciences, USA) onto the plate surface and incubated for 2 hours. 100μl of the cell suspension (1×10^5^ cells) were suspended by RPMI1640 without fetal bovine serum and then added to the upper chamber of each insert which was coated with Matrigel. Next, 500μl RPMI1640 with 10% fetal bovine serum were added into the lower chamber. The cells were incubated for 24-48h to invade in a 5% CO_2_ humidified incubator. After incubation, the cells were washed by 95% alcohol and stained with crystal violet. The cells on the upper surface were removed by cotton swab and the cells which invaded the bottom surface were counted and imaged by microscope at ×200 magnification over four random fields each well. And each experiment was performed in triplicate.

### Tumorigenicity *in vivo*

All animal experiments were approved by the NJMU Institutional Animal Care and Use Committee. A total of thirty six nude mices (BALB/c nude mice, Vitalriver, Nanjing, China; 4 weeks old) were randomly divided into six groups. SGC-7901-NC, SGC-7901-miR-let-7a inhibitor, SGC-7901-pre-miR-let-7a, BGC-823-NC, BGC-823-miR-let-7a inhibitor and BGC-823-pre-miR-let-7a stably transfected cells were inoculated subcutaneously into the flank of nude mice. Tumors were measured with vernier calipers every 4 days, and the mice were euthanized after 3 weeks. The volume of the implanted tumor was calculated by using the formula: volume = (width^2^ × length)/2. Real-time PCR was used to quantify the level of miR-let-7a in tumors. The expression of PKM2 in tumors was quantified by western blotting.

### Statistical analysis

Every experiment was repeated at least three times. All data was performed using SPSS 22.0 and presented as mean ± standard deviation (SD) or indicated. The date was analyzed using two-tailed Student's *t*-test for the average differences. The expression data for paired samples was analyzed with Wilocoxon rank text. P values less or equal than 0.05 which was considered to be statistically significant.
